# An Organic Chemist’s Guide to Electrospray Mass Spectrometric Structure Elucidation

**DOI:** 10.3390/molecules24030611

**Published:** 2019-02-10

**Authors:** Arnold Steckel, Gitta Schlosser

**Affiliations:** 1 Hevesy György PhD School of Chemistry, ELTE Eötvös Loránd University, Pázmány Péter sétány 1/A, 1117 Budapest, Hungary; starnold@caesar.elte.hu; 2 Department of Analytical Chemistry, ELTE Eötvös Loránd University, Pázmány Péter sétány 1/A, 1117 Budapest, Hungary

**Keywords:** tandem mass spectrometry, MS/MS fragmentation, collision-induced dissociation, CID, ESI, structure elucidation

## Abstract

Tandem mass spectrometry is an important tool for structure elucidation of natural and synthetic organic products. Fragmentation of odd electron ions (OE^+^) generated by electron ionization (EI) was extensively studied in the last few decades, however there are only a few systematic reviews available concerning the fragmentation of even-electron ions (EE^+^/EE^−^) produced by the currently most common ionization techniques, electrospray ionization (ESI) and atmospheric pressure chemical ionization (APCI). This review summarizes the most important features of tandem mass spectra generated by collision-induced dissociation fragmentation and presents didactic examples for the unexperienced users.

## 1. Introduction

Electron ionization (EI), a hard ionization technique, is the method of choice for analyses of small (<1000 Da), nonpolar, volatile compounds. As its name implies, the technique involves ionization by electrons with ~70 eV energy. This energy is high enough to yield very reproducible mass spectra with a large number of fragments. However, these spectra frequently lack the radical type molecular ions (M^+^) due to the high internal energy transferred to the precursors [1]. This possible lack of molecular weight information is one of the greatest limitations of this ionization method.

Soft ionization techniques, including electrospray ionization (ESI) and atmospheric pressure chemical ionization (APCI) are nowadays routinely used to ionize thermally labile and at least moderately polar organic analytes. These methods usually yield ions with no unpaired electrons (even-electron ions, EE) and the resulting [M + H]^+^ species are referred to as protonated molecules. Note that use of the term quasi-molecular ions is discouraged [2]. These ions possess low internal energy, therefore fragmentation in a single stage MS experiment is negligible [3,4,5,6,7,8]. This way, the intact accurate mass for sensitive, fragile compounds and large biomolecules as well as their detailed structures could be determined, even when high pressure liquid chromatography is used as a prefractionation method. As the application of electrospray ionization is far more widespread than any other ionization type, only this technique will be reviewed herein.

## 2. Choosing the Precursor Ion for Fragmentation

ESI methodology can be used in either positive (ES+) and negative ion mode (ES−), depending on the proton affinity of the compound. In ES+, only analytes with at least one positive elementary charge, and in ES−, only analytes with at least one negative elementary charge, can be detected. Molecules possessing basic characteristics could be easily ionised in ES+ via making adducts with proton(s), while molecules having acidic functional groups—and lacking basic ones—typically give better spectra in ES−. Optimal ionization mode selection can be based on the Brønsted-Lowry acid-base theory [9]. It should be emphasized that only electrospray-compatible solvents (e.g., acetonitrile, water, methanol, ethanol)—containing 0.1% formic or acetic acid to enhance protonation—or solvent mixtures can be used for dissolving samples during sample preparation. Solvents are highly recommended to be of LC-MS grade. Doubly distilled water should be used for aqueous solutions to effectively minimize the intensity of sodium adducts in the spectra. It is also advisable to use low-binding, high-quality plastic sample tubes to decrease the otherwise always present plasticizer contamination.

The type of EE ions generated in the source are usually singly or multiply protonated [M + *z*H]*^z^*^+^ ions in ES+, while singly deprotonated [M − H]^−^ ions are dominant in ES−. Multiply protonated species are usually formed from large molecules (e.g., polymers), or ones having multiple favorable sites for protonation. These ions are fragmented more easily due to charge repulsion; however, fragments are often not too informative. If the molecule is large enough, it will be represented by an envelope-shaped distribution of peaks.

**Figure 1:**
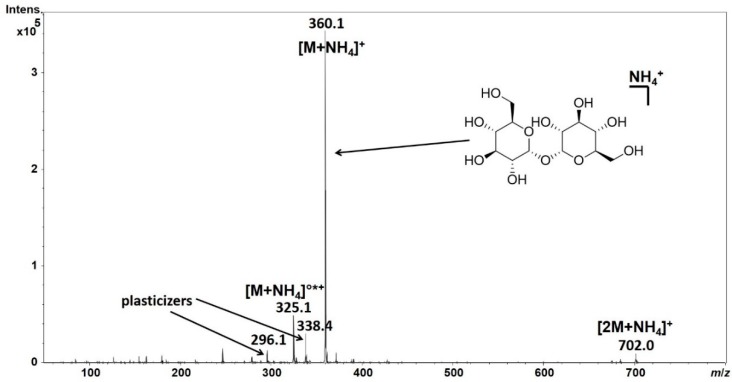
Single stage mass spectrum of trehalose, a disaccharide found e.g., in insects. Due to the lack of basic residues in the molecule, [M + H]^+^ ions could not be detected if the sample was dissolved in acetonitrile-water 1:1 (*v*/*v*) solvent mixture, containing 0.1*%* acetic acid (spectrum not shown). However, using 5 mM of ammonium acetate in water resulted in the above spectrum. Although absolute intensity is still low, the ammonium adduct of trehalose and its cationized dimer could also be detected. Also, a possible in-source fragmentation product can be seen at *m*/*z* 325 displaying a neutral loss of water (°) and ammonia (*). Note the peaks corresponding to *m*/*z* 296.1 and *m*/*z* 338.4 are very likely the [M *+* NH_4_]^+^ ion of dibutyl phtalate and [M + H]^+^ ion of erucamide, respectively, which are common plasticizers leaked from plastic sample tubes.

Charge distributions can be altered by using buffers. Adduct formation could also take place depending on the Lewis basicity of the compound in the presence of NH_4_^+^, Na^+^ and K^+^ or if Li^+^ or Ag^+^ is intentionally added to the sample [10,11]. Note that these ions should be referred to as cationized molecules as the terms quasi-molecular ion and pseudo-molecular ion are discouraged [2]. These adduct ions usually give no fragmentation other than the loss of the cation, but for special compounds (e.g., carbohydrates), their fragmentation could be also significantly different from that of [M + H]^+^ ions, giving useful structural information [12]. Ammonium acetate or bicarbonate are usually used for adduct formation typically in 5 mM concentration (Figure 1). It is worth noting that additives with high ionization efficiency (e.g., tertiary or quaternary amines) and ones causing significant suppression of signal intensity (e.g., phosphate buffers, sodium chloride, trifluoroacetate) should be avoided. In ES−, adduct formation could also happen; anionized molecules may be useful in the detection of some compounds (e.g., carbohydrates, flavonoids).

**Figure 2:**
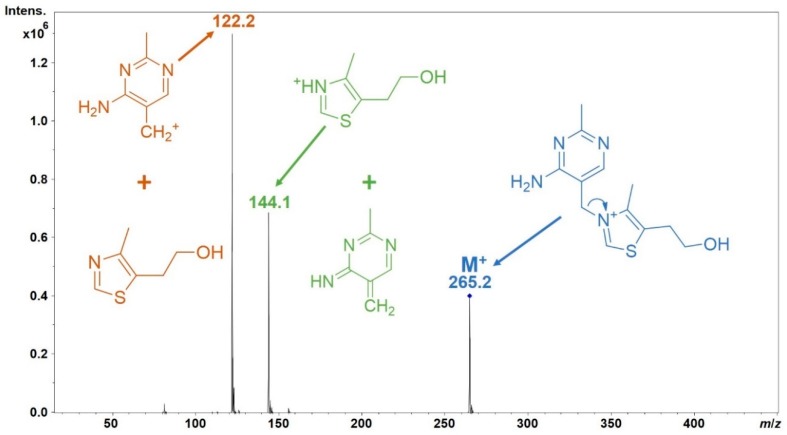
Illustration of Field’s rule on thiamine (vitamin B_1_) in positive mode (ES+). Note that the precursor in blue is not a protonated thiamine molecule [M + H]^+^, but simply an M^+^, as the compound intrinsically has a fixed positive charge. Upon collision-induced dissociation, it decomposes via two different pathways (orange and green colors) to yield product ions (upper structures) and neutral molecules (lower structures). The pathway in orange represents an inductive cleavage (see also Scheme 1), while the one in green indicates an acyclic hydrogen rearrangement competitive with the former. Only charged fragments could be detected (upper structures), and their intensities are proportional to their neutral forms’ proton affinity. Note that the fragments could be represented with multiple other (more stable) resonance structures; here, only one of each is depicted due to didactic reasons.

If the analyte is prone to complex formation or is highly concentrated, [2M + H]^+^ or [2M − H]^−^ ions could also be present in the full mass spectra. It is also common to detect M^+^ ions using soft ionization methods if the compound already had a fixed charge before ionization (Figure 2). Typical examples are quaternary ammonium salts. The distribution of charge via conjugation also enhances the stability of the protonated/deprotonated ion as e.g., in case of compounds with a dienone moiety. If a given compound (e.g., ketones, aldehydes, alcohols) cannot be detected in either mode or fragmentation efficiency needs to be enhanced, one could use derivatization agents [13]. These compounds are usually basic to enhance ionization efficiency. The limitation of this approach is that it cannot be used for totally unknown compounds. Another way is to use a different ionization type such as APCI [14] or atmospheric pressure photoionization (APPI), which may be more applicable for less polar compounds (e.g., lipids) [15]. Note that molecular ions (M^+^) could be also observed using APCI or APPI [14,15,16].

## 3. Performing MS/MS Experiments

To obtain structural information from an EE ion, an MS/MS (tandem mass spectrometric) experiment is needed either in a collision cell or in an instrument featuring an ion trap analyzer. Protonated initial molecules selected for fragmentation are called precursor ions, while their fragments are referred to as product ions. The terms mother and daughter ions are discouraged [2], however, they are still widely used. Both in traps and collision cells, the analyte ions generated by the ion source are excited by collisions with inert gases e.g., N_2_, Ar or He—depending on the instrument type—to impart enough energy inducing decomposition processes [17]. It should also be noted that fragmentation in the ion source could occur with considerable extent for fragile compounds or by altering source parameters. This could be exploited in case of using quadrupole ion traps where useful fragments could be missing in standard MS/MS spectra due to a technical limitation called low-mass cut-off. Another way for the induction of in-source fragmentation where there is no second mass analyser (e.g., single quadrupole instruments) is performing cone voltage fragmentation/declustering potential fragmentation. These methods could only be used however if the compound is purified or MS is hyphenated with HPLC. Investigation of the fragmentation processes in case of EE ions are usually carried out using various methods, such as theoretical calculations, stable isotope labelling, H/D exchange [18] and MS^n^ experiments on instruments capable of high resolution and high mass accuracy (HRAM-MS/MS). Orbitrap and Q-TOF instruments are usually the best method of choice for these purposes as these are the most widely applicable types. When calculating molecular masses and comparing them to experimentally obtained values, monoisotopic masses (often called exact masses in molecule editor programs) must be used if the instrument is capable of HRAM-MS/MS. If MS/MS is carried out in TOF/TOF instruments (keV energies imparted to precursors) or is coupled to ion mobility spectrometry, discrimination between constitutional isomers or diastereomers respectively, is possible [19,20,21]. These methods are mostly used in the analyses of biomolecules.

In the following, the general rules for fragmentation processes of protonated molecules will be outlined. The rules will be applied on some examples to demonstrate their usefulness. There is far less information on the fragmentation processes of deprotonated molecules in the literature, so we will restrict the observations for ES+ mode. One reason for this is that the formation of deprotonated molecules is usually limited to compounds possessing acidic protons or compounds that could generate such ones by tautomerism. Other reasons include the poor understanding of fragmentation processes and technical difficulties. The signal intensity for ES− is usually lower, therefore whenever possible, use of ES+ is recommended. However, ES− is of high importance e.g., for the characterization of flavonoids [6], oligosaccharides [22], carboxylic acids [23,24], sulfonamides [25], oligonucleotides [26] and rarely peptides [27].

## 4. General Fragmentation Characteristics of Protonated Molecules

The fragmentation characteristics of an [M + *z*H]*^z^*^+^ type protonated molecule and an M^+^ radical type molecular ion is significantly different (Table 1).

OE ions tend to fragment at more random sites, while even-electron ions usually produce less but more stable fragments by cleavages at the thermochemically least stable bonds. It is also possible to form an odd-electron ion from an ESI-generated multiply charged even-electron ion precursor with methods of electron capture dissociation (ECD) [29] or electron transfer dissociation (ETD) [30] which nowadays have widely-used application in sequencing of peptides and other biomolecules.

It is supposed that the mobile proton hypothesis could also play an important role in the fragmentation of protonated small organics, as previously described for peptides in the literature [31,32]. Based on this hypothesis, protons occupy the favored basic sites of the molecules via soft ionization in the source. Afterwards, excitation in the collision cell or in the trap results in proton migration throughout the structure. When protons reach a specific position causing thermal instability for the system, the ion decomposes leading to two fragments: one of them will carry the original charge, the other will be eliminated as a neutral molecule.

According to some authors, the neutral molecule and the charged ion could also form an ion/neutral complex [33] and the to-be-fragments could successfully compete for the sequestration of the proton yielding to complementary fragment ions in the MS/MS spectra. The most important qualitative rule to predict which fragment will retain the charge and how abundant will be in the spectrum is to investigate the proton affinity of their neutral counterparts. The higher is the proton affinity of a neutral, the higher is the intensity of its charged form in the spectrum. This is called Field’s rule [34] and is depicted in Figure 2.

All these processes are unimolecular; therefore, fragments could only contain elements that could already be found in the precursor as well. The presence of elements with special isotope distribution like bromine, chlorine, certain transition metals or intentionally introduced stable isotopes could also contribute to an easier structure elucidation.

The so-called ‘nitrogen rule’ is routinely applied for EI spectra. It states that a singly charged OE precursor/fragment ion with an odd nominal *m*/*z* contains odd number of N atoms; an ion with an even nominal *m*/*z* contains even number of N atoms. The nitrogen rule is also applicable for soft ionization methods [1,28] (Table 2): an odd nominal *m*/*z* of an EE ion indicates an even number of N atoms, and an even nominal *m*/*z* of an EE ion indicates an odd number of N atoms.

### Types of Cleavages and Reaction Routes

The fundamental fragmentation pathways could be classified as radical site-induced fragmentation, charge-induced fragmentation and charge-remote fragmentation according to McLafferty [1]. For odd-electron ions, all these routes are allowed, in contrast to even-electron ions where only the last two are possible. For depiction of mechanisms, curved double-headed arrows are used for the movement of an electron pair while single-headed ones are for displaying the motion of a single electron.

**Scheme 1:**
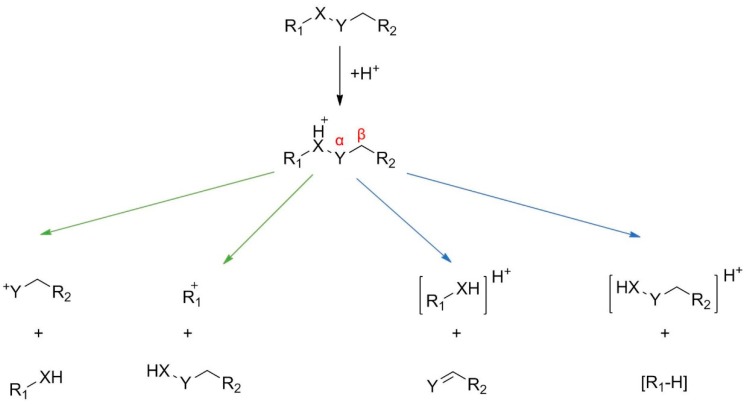
Representation of the main noncyclic fragmentation pathways characteristic to even-electron ion fragmentation in ES+ mode. Green arrows indicate inductive cleavages, while blue ones denote acyclic β-*H*-rearrangements. The molecule of interest must contain at least one functional group prone to be protonated. X denotes -NH, -O- or -S- groups, while Y denotes -CH_2_-, C=O, -SO_2_ ones so that the general fragmentation routes for amines, (sulfon)amides, ethers, esters, thioesters are given here. Molecules featuring groups not covered by the scheme above, like alcohols or carboxylic acids are prone to lose water or carbon dioxide/formic acid upon collision-induced dissociation respectively. Ketones usually dissociate to yield acylium ions, while simple aldehydes are often cannot be detected without derivatization. Expressive and more specific examples are collected by Niessen et al. [2] (p. 84; or 94–101 for general routes of fragmentation for specific compound classes).

**Figure 3:**
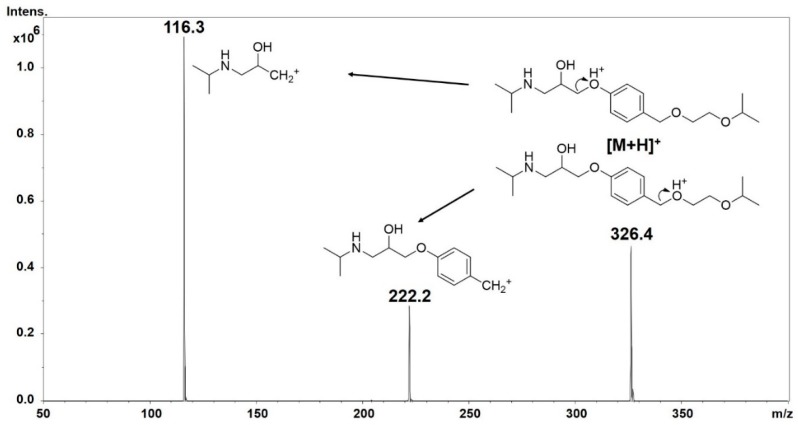
Tandem mass spectrum of bisoprolol, a cardioselective β-blocker. The initial site of protonation is the basic sp^3^ N. Without this moiety, the molecule cannot be detected. After collision-induced activation, the precursor ion [M + H]^+^ can be represented by two structures. All two fragments of the precursor ion are a result of inductive cleavages in concordance with Scheme 1, green arrows (in this case, X = O, Y = CH_2_). Note that these fragments not necessarily represent the most stable resonance structures.

**Figure 4:**
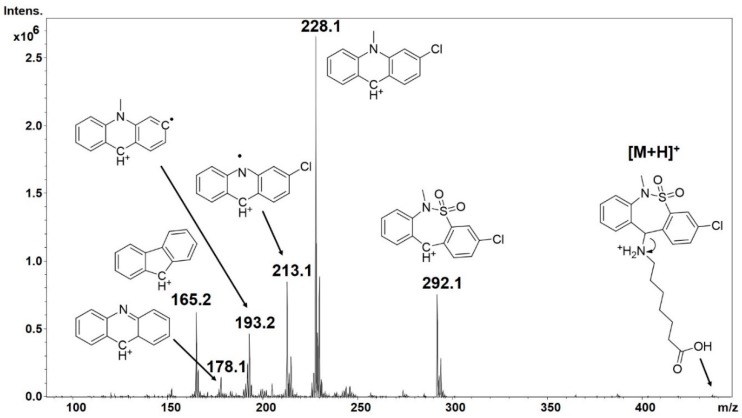
In-source fragmentation of protonated tianeptine, marketed as a nootropic ‘dietary supplement’ in the USA. Note that *m*/*z* 213 and 193 correspond to fragments that violate the above-mentioned even-electron rule. Elimination of SO_2_ is a charge-remote neutral loss characteristic to sulfonamides [38], sulfonic acids and aromatic sulfoxides. The mechanisms for losses and rearrangement reactions are often not trivial but rules known from organic chemistry regarding stability of ions could be successfully applied.

**Figure 5:**
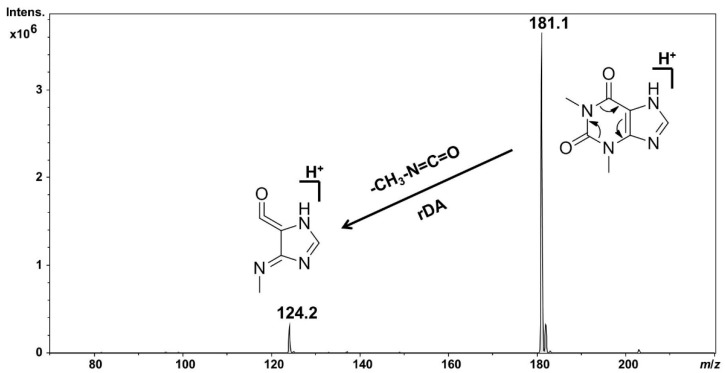
Representation of retro-Diels-Alder reaction (rDA), a typical charge remote process on protonated theophylline. The neutral molecule eliminated here is a characteristic loss for other xanthines as well. The name charge remote reflects that the reaction takes place without the involvement of the ionizing proton. Therefore, the exact site of ionizing proton is usually not depicted.

**Figure 6:**
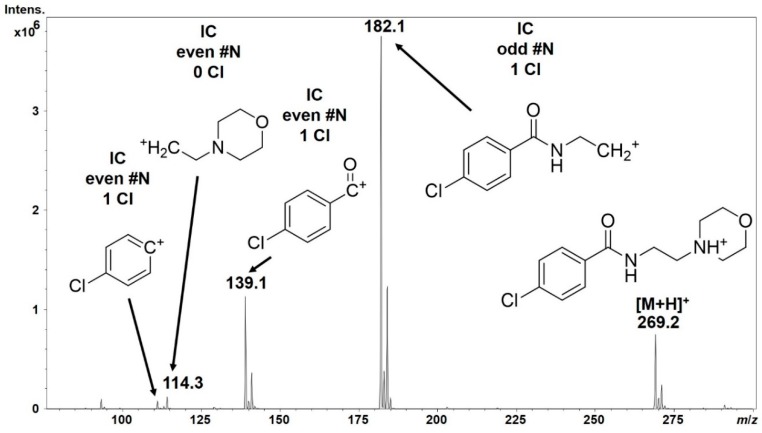
The application of all rules concerning fragmentation on the example of protonated moclobemide, a reversible monoamine oxidase inhibitor. IC stands for inductive cleavages and all the fragments could be explained by this route alone. There is no sign of violation of the even-electron rule. Note the characteristic isotope pattern which could be attributed to a single chlorine atom in the precursor and some of the fragments. An even *m*/*z* value (rounded to integer) implies an uneven number of nitrogens in the given ion and vice versa for an even-electron ion which is in concordance with the nitrogen rule.

Bond cleavages upon the decomposition of an ion can be sorted as either homolytic or heterolytic. Another type of characterization of the fragmentation processes is based on the retention of the charge. In case of charge retention fragmentations (CRF) the charge is retained on the atom possessing the charge initially, while in case of charge migration fragmentations (CMF) charge is transferred to one of the atoms in the other fragment. The most fundamental fragmentation pathways of positively charged even-electron ions are summarized in Scheme 1. For more specific examples, see Figure 3, Figure 4, Figure 5 and Figure 6 (from the authors’ own works) or Niessen et al. [2,35].

Fragmentation of even-electron ions usually follow the so-called even-electron rule [36,37]. Based on the even-electron rule, the formation of ions with no unpaired electrons are favored upon fragmentation, along with loss of neutral molecules from the precursor, instead of homolytic cleavages which are rare amongst this type of ions. Eliminations of neutral molecules could be characteristic (selective or specific) to certain types of compounds. For a very thorough and useful list on characteristic neutral losses, see Niessen et al., 2017 [2].

Heterolytic cleavage is often referred to as inductive cleavage and can be observed for both OE and EE ions. The inductive cleavage of the carbon-heteroatom bond is a CMF leading to an EE fragment. Typical inductive cleavage products are represented in Figure 3 for protonated bisoprolol. A competitive fragmentation pathway with inductive cleavage of EE ions is the acyclic β-*H*-rearrangement (a CRF) and could be deducted from an inductive cleavage where a proton at the β-carbon migrates to the other fragment. Thus, in soft ionization, heterolytic cleavage also include acyclic β-*H*-rearrangement. For both pathways, the sum of the two fragments is equal to M + 2H^+^, and the relative abundance of them is determined by the Field’s rule. Inductive cleavage and acyclic β-*H*-rearrangement are two of the most important cleavage types in the dissociation of EE^+^ ions.

Homolytic cleavages include σ- (mostly for OE) and α-cleavage (only for OE). The role of σ-bond homolytic cleavage in EE+ fragmentation processes are limited to certain types of compounds having a carbon-heteroatom bond resulting in the loss of radical species such as Cl˙, NO_2_˙, ˙CH_3_, CH_3_O˙ [3,27]. For this type of violation of even-electron rule, see Figure 4. These losses of radicals are usually competitive with the loss of their non-radical counterparts e.g., HCl.

It is often required for atoms and bonds to be rearranged either through a six-membered cyclic transition state or through ion/neutral complexes prior to fragmentation. Most common rearrangement types for protonated molecules include retro (hetero)ene reactions and retro Diels-Alder reaction. Didactic examples for these types are reviewed by Demarque et al. [6]. A retro-heteroene reaction could be considered as the even-electron counterpart of McLafferty rearrangement. In case of retro-Diels-Alder reaction, the six-member ring with a double bond is given in the first place, there is no need for the preformation of such structures. These three reactions along with the elimination of small molecules (e.g., H_2_O, CO, NH_3_) are special types of charge remote fragmentation, where the cleavage is unrelated to the location of charge. The retro-Diels-Alder reaction (rDA) is also known from chemical synthesis. As opposed to OE, in the case of EE ions, the process is most probably not radical initiated, and involves electron pair migrations in the ring. This type of fragmentation can be used e.g., for the structure elucidation of flavonoids [39,40]. A typical rDA reaction is shown for theophylline (a xanthine) in Figure 5. The rules introduced above are successfully applied to identify the possible fragments of moclobemide shown in Figure 6.

## 5. Software-Aided Structural Analyses

The above-mentioned selected examples could be very useful for novices to understand the basics of ESI-MS spectra evaluation. When analyzing compounds possessing multiple functional groups and/or condensed ring systems however, evaluation of MS/MS spectra could be very complicated and interpretation of these spectra is challenging even for experts (see Figure 4). In these cases, software-based spectra predictors or compound identifiers serve as a good alternative. A user-friendly and free to download software with free source code for these purposes could be found for example on the website http://cfmid.wishartlab.com/ [41]. These work by algorithms based on experimental MS/MS spectral databases. Even in these cases, proposed fragment ion structures could not always be easily elucidated. It is also very often for product ion predictors to fail to explain a significant number of observed peaks in the experimental spectrum, especially for compounds that have not been described previously.

## 6. Summary

Here, we made an attempt to bring the field of electrospray-tandem mass spectrometry closer to organic chemists unexperienced in this field. Key pillars of the evaluation of tandem mass spectra of organic compounds can be summarized in the following points:

- selection of ionization mode is based on the basicity of the compound of interest
- analytes are ionised to yield even-electron ions in most cases
- the fragmentation processes of even-electron ions are much closer to the concepts laid down in organic chemistry as opposed to the ones observed in electron ionization
- the resulting fragments are generally even-electron ions
- elimination of small neutral molecules from precursors are preferable
- most fragment ions are a result of inductive cleavages, acyclic hydrogen rearrangements as well as some (a)cyclic special rearrangements
- the identity of a previously described compound in an unknown sample could be determined by using (U)HPLC-ESI-MS/MS and software-based annotation

## 7. Notes

All the MS(/MS) spectra are the own work of the authors and were obtained on an Esquire 3000+ ion trap instrument (Bruker, Bremen, Germany). Pure samples were dissolved in acetonitrile-water (1:1, *v*/*v*) mixture containing 0.1% acetic acid unless otherwise stated.

## Figures and Tables

**Table 1:** Comparison of the fragmentation of odd and even-electron precursors upon fragmentation.

	Odd Electron Precursor (OE)	Even-Electron Precursor (EE)
**Ionization**	EI	ESI, MALDI, APCI, (APPI) ^1^
**Type of precursor**	M^+^	[M + *z*H]*^z^*^+^, [M + C]^+^, [M − H]^−^, [M + A]^−^
**Nitrogen rule**	✓	✓
**RDBE ^2^**	integer	half-integer
**Type of fragments**	OE	EE
**Prediction of fragment abundance**	Stevenson’s rule	Field’s rule
**Preferred cleavage type**	homolytic	heterolytic

^1^ ESI: electrospray ionization, MALDI: matrix-assisted laser desorption/ionization, APCI: atmospheric pressure chemical ionization, APPI: atmospheric pressure photoionization. ^2^ RDBE: ring and double bond equivalent. RDBE should be calculated as $N_{X}-\frac{N_{Y}}{2}\;+\;\frac{N_{Z}}{2}\;+\;1$ where X = C, Si; Y = H or halogens, Z = N, P ([1], p. 28, [28]) Note that sulfur and oxygens atoms are not included in this formula and all atoms are considered with their lowest valence state. Despite other attempts to calculate the degree of unsaturation, there is none that could be used without limitation.

**Table 2:** The extension of nitrogen rule to even-electron ions ([1], p. 38). Note that the N rule is only applicable for singly charged ions containing solely H, C, N, O, Hlg, P and S atoms and is only reliable < *m*/*z* 500 [28].

	Odd *m*/*z*	Even *m*/*z*
OE ^1^	odd #N	even #N
EE ^2^	even #N	odd #N

^1^ odd-electron precursor/fragment ion. ^2^ even-electron precursor/fragment ion.
